# Amelioration of ocean acidification and warming effects through physiological buffering of a macroalgae

**DOI:** 10.1002/ece3.6552

**Published:** 2020-07-19

**Authors:** Steve S. Doo, Aero Leplastrier, Alexia Graba‐Landry, Januar Harianto, Ross A. Coleman, Maria Byrne

**Affiliations:** ^1^ Coastal & Marine Ecosystems Group School of Life & Environmental Sciences The University of Sydney Sydney NSW Australia; ^2^ Geoecology and Carbonate Sedimentology Group Leibniz Centre for Tropical Marine Research (ZMT) Bremen Germany; ^3^ Research School of Earth Sciences The Australian National University Canberra ACT Australia; ^4^ ARC Centre of Excellence for Coral Reef Studies James Cook University Townsville Qld Australia

**Keywords:** large benthic foraminifera, macroalgae, ocean acidification, ocean warming, physiological buffering, species interaction

## Abstract

Concurrent anthropogenic global climate change and ocean acidification are expected to have a negative impact on calcifying marine organisms. While knowledge of biological responses of organisms to oceanic stress has emerged from single‐species experiments, these do not capture ecologically relevant scenarios where the potential for multi‐organism physiological interactions is assessed. Marine algae provide an interesting case study, as their photosynthetic activity elevates pH in the surrounding microenvironment, potentially buffering more acidic conditions for associated epiphytes. We present findings that indicate increased tolerance of an important epiphytic foraminifera, *Marginopora vertebralis*, to the effects of increased temperature (±3°C) and *p*CO_2_ (~1,000 µatm) when associated with its common algal host, *Laurencia intricata*. Specimens of *M. vertebralis* were incubated for 15 days in flow‐through aquaria simulating current and end‐of‐century temperature and pH conditions. Physiological measures of growth (change in wet weight), calcification (measured change in total alkalinity in closed bottles), photochemical efficiency (*Fv/Fm*), total chlorophyll, photosynthesis (oxygen flux), and respiration were determined. When incubated in isolation, *M. vertebralis* exhibited reduced growth in end‐of‐century projections of ocean acidification conditions, while calcification rates were lowest in the high‐temperature, low‐pH treatment. Interestingly, association with *L. intricata* ameliorated these stress effects with the growth and calcification rates of *M. vertebralis* being similar to those observed in ambient conditions. Total chlorophyll levels in *M. vertebralis* decreased when in association with *L. intricata*, while maximum photochemical efficiency increased in ambient conditions. Net production estimates remained similar between *M. vertebralis* in isolation and in association with *L. intricata*, although both production and respiration rates of *M. vertebralis* were significantly higher when associated with *L. intricata*. These results indicate that the association with *L. intricata* increases the resilience of *M. vertebralis* to climate change stress, providing one of the first examples of physiological buffering by a marine alga that can ameliorate the negative effects of changing ocean conditions.

## Graphical abstract

We present findings that indicate increased tolerance of an important epiphytic foraminifera, *Marginopora vertebralis*, to the antagonistic effects of increased temperature and *p*CO2, when associated with its common algal host, *Laurencia intricata*. When incubated in isolation, *M. vertebralis* exhibited reduced growth in end‐of‐century projections of ocean acidification conditions, while calcification rates were lowest in the high‐temperature, low‐pH treatment. Interestingly, association with *L. intricata* ameliorated these stress effects with the growth and calcification rates of *M. vertebralis* being similar to those observed in ambient conditions.

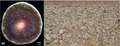

## 1 INTRODUCTION

Increased anthropogenic CO_2_ has caused physical and chemical changes to oceans worldwide causing global climate change (GCC; warming; IPCC, 2013) and ocean acidification (OA; Caldeira & Wickett, 2003). These physical and chemical changes are expected to have severe impacts on marine biota and in particular organisms that produce calcium carbonate shells or tests (Byrne & Fitzer, 2019; Kroeker et al., 2013; Kroeker, Kordas, & Harley, 2017). Although most studies to date have focused on single‐species responses to climate change stressors (reviewed in Byrne & Fitzer, 2019; Hofmann et al., 2010; Przeslawski, Byrne, & Mellin, 2015), recent work highlights the importance of considering multispecies interactions in future multistressor ocean conditions (Kroeker et al., 2017). These interactions, framed in the context of ecological theory, provide relevant and more accurate predictions of how organisms will respond to climate change stress (Gaylord et al., 2015), and nuanced aspects of responses to climate change are overlooked in single‐species studies. Examples of compensatory mechanisms whereby interactions between multiple organisms modify organism physiology have been shown to buffer against stress to maintain ecological equilibria (Doo, Carpenter, & Edmunds, 2018; Ghedini, Russell, & Connell, 2015). In particular, metabolic processes, such as photosynthesis in primary producers, have the potential to ameliorate the negative effects of projected climate change conditions (e.g., OA) through physiological interactions between multiple species (Connell et al., 2017).

On coral reefs, ecological interactions in the form of symbiosis (mutualism, parasitism, commensalism) between organisms drive high diversity (Hughes et al., 2003). Endosymbiotic marine relationships (and the transfer of energy from the endosymbiont to the host) have been relatively well studied in corals (e.g., Little, van Oppen, & Willis, 2004), but the effects of climate change on epibiont and ectosymbiont relationships are largely unknown. Macroalgae present an interesting case study, as they produce a diffusive boundary layer (DBL) of increased pH due to photosynthesis (Cornwall, Hepburn, Pilditch, & Hurd, 2013; Hurd et al., 2011). These layers have been observed on the micro‐scale and have been hypothesized to buffer against the effects of ocean acidification on macroalgae and in seagrass beds (Bergstrom, Silva, Martins, & Horta, 2019; Cornwall et al., 2013; Hurd et al., 2011), although the corresponding increase in respiration can potentially negate these positive effects (Kapsenberg & Cyronak, 2019). Although macroalgal DBLs should influence the physiology of their calcifying epiphytes, observations at CO_2_ seeps in Papua New Guinea and Mexico have shown negligible effects of buffering by seagrasses on calcifying epibionts in response to decreasing pH (Fabricius et al., 2011; Pettit, Smart, Hart, Milazzo, & Hall‐Spencer, 2015). In contrast, a recent study identified increased abundance of mollusk species in turf algae at a temperate CO_2_ seep site compared with adjacent ambient sites, suggesting that turf algae provide large DBLs, which benefit calcifying organisms residing on that substrata (Connell et al., 2017).

**FIGURE 1:**
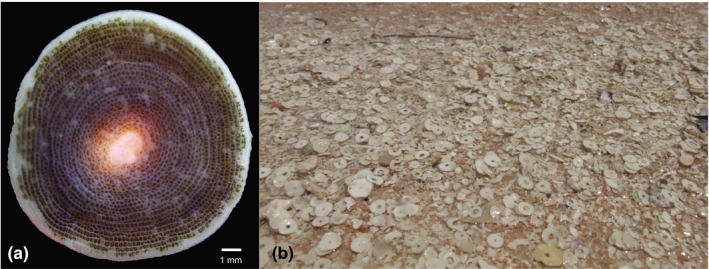
Photograph of (a) *Marginopora vertebralis* taken using light microscopy. The dark green color indicates the presence of *Symbiodinium* sp. microsymbionts. Scale bar is 1 mm. (b) Postmortem, *M. vertebralis* is important in beach sand production, as seen in the foreground of the photograph, where white tests are seen. This image was taken at Coconut Beach, Lizard Island Reef, Australia

Foraminifera are single‐celled organisms that reside in oceans worldwide, serving as a major carbon sink in both pelagic and benthic marine habitats (Langer, 2008). Shell geochemistry of planktic foraminifera has been extensively used as paleoindicators of past ocean conditions and climactic change (Hönisch et al., 2012; Spero, Bijma, Lea, & Bemis, 1997). Recently, anthropogenic‐driven alterations in ocean chemistry have been identified in planktic foraminifera through changes in population density and shell thickness (Moy, Howard, Bray, & Trull, 2009; Osborne, Thunell, Gruber, Feely, & Benitez‐Nelson, 2020). Benthic species, specifically large benthic foraminifera (LBFs), often cohabit with macroalgal substrata in shallow‐water coral reef flats where they benefit from this symbiosis (Doo, Hamylton, & Byrne, 2012). Many LBFs such as including *Marginopora vertebralis* form symbioses with marine microalgae (Figure 1a). These protists are especially important in terms of biogeochemical processes of carbon sequestration and generation of biogenic calcium carbonate (Langer, 2008; Langer, Silk, & Lipps, 1997). LBFs generate >95% of carbonate sands in some areas of coral reefs (Figure 1b; Baccaert, 1987; Davies & West, 1981) and up to 4%–6% of total carbonate storage of certain coral reef cays (Doo, Hamylton, Finfer, & Byrne, 2016). Recent studies on LBFs have showed varied but generally negative results of calcification and growth when exposed to increasing OA and GCC (reviewed in Doo, Fujita, Byrne, & Uthicke, 2014). Previous work on *M. vertebralis* has found increased productivity in associated dinoflagellate symbionts in response to near‐future OA conditions, but this did not compensate for increased metabolic demands, and ultimately resulted in lower calcification rates for this species when compared to ambient conditions (Naidu, Hallock, Erez, & Maata, 2017; Uthicke & Fabricius, 2012).

To date, nearly all climate change studies of LBFs have involved experiments with these organisms in isolation, often not representative of natural systems. Most tropical LBFs occur in high densities associated with corticated and calcareous marine macroalgae such as *Laurencia* spp. and *Halimeda* spp. (Langer, 1993). In this study, we explore the potential for the noncalcifying macroalgae *L. intricata* to buffer *M. vertebralis* against the negative effects of acidification and warming. Macroalgae and LBFs were incubated in flow‐through experiments that manipulated seawater conditions to simulate current (control) and end‐of‐century projected ocean conditions. Physiological variables of calcification, growth rate, photochemical efficiency, respiration, and total chlorophyll were quantified to assess how the exposure of macroalgae affected *M. vertebralis* physiology. We tested three hypotheses: (a) Similar to previous experiments, in isolation, *M. vertebralis* would exhibit decreased growth and calcification in response to OA and GCC, but (b) the negative effects of acidification and warming would be ameliorated when in association with *L. intricata,* and 3) this association elicits a compensatory mechanism, whereby increased metabolic parameters (photosynthesis) modulate calcification when *M. vertebralis* is in association with *L. intricata*.

## 2 MATERIALS AND METHODS

### 2.1 Collection and acclimation

Specimens of *M. vertebralis* (as identified by Renema, 2018) and *Laurencia intricata* were collected from Coconut Beach (1–3‐m depth), Lizard Island (014°40′08″S, 145°27′34″E) on the Great Barrier Reef, Australia, in October 2015 (Figure 1). Samples were immediately transported back to Lizard Island Research Station and placed into flow‐through ambient seawater conditions and light for 5 days to acclimate to laboratory conditions. Specimens of *L. intricata* were then separated into ~1 g (wet weight) replicates, and all visible epiphytes (*M. vertebralis* and other LBFs such as *Calcarina hispida, Amphistegina lobifera*, and *Baculogypsina sphaerulata*) were removed. The *M. vertebralis* were separated into experimental replicates in which 6 *M. vertebralis* (~0.5 g wet weight, all approximately similar size of ~5‐mm diameter) were placed into 60 ml jars with 40 ml of seawater, similar to densities found in situ (Doo, pers. obs.). The experimental treatment groups of *M. vertebralis* only and *L. intricata* with *M. vertebralis* were established prior to the start of the experiment and acclimated 3 days before the initiation of the experiment. Specimens were incubated in polypropylene jars with a hole cut from the side to prevent overflow over the top. A 462‐µm plankton mesh was glued to the side of the jar to allow for overflow of water through the mesh, while maintaining flow‐through conditions, and resulted in a total of ~40 ml water in each container. Light was provided using LED cool white lights (LED type 3528) to an intensity of ~100 µmol photons m^−2^ s^−1^ for the duration of the experiment (2 weeks) in a 12 hr:12 hr, day–night cycle. A flow‐through dripper tap system was used for experimental water delivery (~40 ml/min). Experimental conditions were gradually reached over a 3‐day period, with an increase of 1°C and decrease of 0.1 pH unit each day prior to the start of the experiment when all replicates were incubated in ambient temperature (~25.5°C and ~pH_Total_ 7.95 conditions). Experimental water was collected from Lizard Island lagoon, filtered with a 5‐µm filter bag, and delivered into 60 L header tanks, from which all treatment groups were supplied incubation water.

Physicochemical conditions of two temperatures (ambient [26°C] and high [29°C]), and two pH's (ambient [8.0] and low [7.7] pH_Total_ units) in a fully orthogonal treatment group design was used. To determine the effect of symbiosis of *L. intricata* and *M. vertebralis*, 10 replicates each of *M. vertebralis* only and *L. intricata* with *M. vertebralis* were incubated in each of the pH/temperature treatment groups for a total of 80 replicates.

### 2.2 Incubation parameters and seawater chemistry

The seawater pH and temperature conditions were controlled using a Neptune Apex system dosing pure CO_2_ to regulate pH. In the experiment, a total of 4 sumps were used, one for each manipulated seawater condition (see above). This water was pumped into individual jars (see above), maintaining independence between replicates. Total alkalinity, pH, and temperature of the header tanks were measured on a daily basis, from randomly selected drippers. Total alkalinity samples were filtered with a 0.22‐µm filter prior to analysis to eliminate possible contamination of calcium carbonate in the sample and measured using open‐cell potentiometric titrations (Dickson, Sabine, & Christian, 2007). Seawater pH was monitored using m‐cresol spectrophotometric measurements on an Ocean Optics USB4000+ spectrophotometer, and pH_Total_ calculated based on standard protocols (Dickson, Sabine, & Christian, 2007). These were referenced to seawater Certified Reference Material (CRM), Batch 161, prepared by A. Dickson in the Scripps School of Oceanography. Temperature and salinity measurements were collected with Vernier TMP‐BTA and CON‐BTA probes, respectively. Seawater parameters remained stable throughout the experimental incubation (Table 1).

**TABLE 1:** Carbonate parameters measured during the experiment

	Ambient temperature		Elevated temperature	
	Ambient pH	−0.3 pH	Ambient pH	−0.3 pH
Temperature (°C; *n* = 30)	25.6 ± 0.3	25.2 ± 0.5	28.1 ± 0.3	28.2 ± 0.5
pH_Total_ (*n* = 30)	7.95 ± 0.01	7.72 ± 0.01	7.95 ± 0.01	7.71 ± 0.01
Salinity (PSU; *n* = 20)	35.2 ± 0.1	35.3 ± 0.1	34.9 ± 0.1	34.9 ± 0.1

Note

Temperature, pH_Total_, and salinity data were collected daily. Data are mean ± *SE*.

### 2.3 Growth measurements

The wet weight of *M. vertebralis* across individual replicates was pooled within replicate jars and measured prior to the start of the experiment and at the termination using a Mettler Toledo ML240 balance to 10^−4^ g resolution. Measurements were then converted into a percentage daily change in weight. In treatments of both *L. intricata* and *M. vertebralis*, only the pooled *M. vertebralis* from each replicate were weighed at the initiation and termination of the experiment.

### 2.4 Instantaneous calcification measurements

After a 2‐week incubation, alkalinity anomaly measurements were made using close bottle experiments as a proxy of instantaneous calcification. Organisms were carefully sealed in ~20 ml glass scintillation vials with their chosen treatment group water, and in the case of the algal associated groups, with the algal hosts. The sealed vials were immersed in the appropriate flow‐through water system to maintain treatment temperature. Treatment groups were incubated for 8 hr in light conditions.

Analyses of water samples for total alkalinity were as above, and calcification (*G*) was calculated using Equation (1): TA is total alkalinity (mmol/kg), m.w. is the molecular weight of CaCO_3_ (100 mg/mmol), *p* is seawater density (1.023 kg/ml), *V* is chamber volume (ml), *w*.*w*. is wet weight of *M. vertebralis* (mg), and *T* is incubation time (d). All calculated values were normalized to final wet weight.

(1) $$G\left({\text{mg CaCO}}_{3}{\;\text{day}}^{‐1}\;\text{mg}\;M.\;vertebralis^{‐1}\right)=‐0.5\times \Delta TA\times m.w.\times \rho \times V\times w.w.^{‐1}\times T^{‐1}$$

### 2.5 Photochemical efficiency measurements

At the termination of the experiment, maximum photochemical efficiency (*Fv/Fm*) data were measured using WALZ DIVING‐PAM underwater fluorometer (similar to Schmidt, Kucera, & Uthicke, 2014). Measurements were recorded 4 hr after sunset in dark conditions, and individual *M. vertebralis*, and averaged across pseudoreplicate *M. vertebralis* within individual treatments.

### 2.6 Total chlorophyll measurements

Following measurement of wet weight, samples were immediately frozen (−20°C) in dark conditions and stored for chlorophyll analyses. Samples were placed in 15 ml polypropylene plastic tubes with 10 ml of 90% acetone and subsequently mechanically ground with a hard metal rod. Samples were then incubated in 4°C overnight in the dark. Absorbance measurements were then taken from the supernatant using an Ocean Optics USB4000+ spectrophotometer, and wavelengths of 630 nm, 647 nm, 664 nm, and 691 nm were recorded. Total chlorophyll was calculated based on universal equations developed by Ritchie (2008). In treatments of both *L. intricata* and *M. vertebralis*, *M. vertebralis* were pooled within the replicate sample jar and measured separately. All measurements were normalized to final wet weight of the corresponding *M. vertebralis* replicate.

### 2.7 Oxygen flux measurements

Oxygen flux measurements were made with a PreSens Oxy‐10 mini 10‐channel optical sensor. At the end of the 15‐day incubation period, oxygen flux measurements were taken in 30 ml glass scintillation vials that were gently stirred. Replicate samples (including *L. intricata* in association treatments) were gently placed in the glass jars with corresponding pH and temperature conditions, and allowed to acclimate for at least 5 min before measurements were recorded. For oxygen production measurements, light conditions in replicates were ~100 µmol photons m^−2^ s^−1^ during measurements (similar to incubation levels) and measured for a total of ~30 min in light conditions first. Subsequently, respiration was measured in dark conditions for ~30 min, allowing for 5 min of acclimation, and rate of oxygen consumption measured after the acclimation period. All analyses were performed using standard protocols for LBFs outlined in Uthicke and Fabricius (2012).

As the association treatment of *M. vertebralis* was incubated with *L. intricata*, an additional set of experiments was performed to separate the effect of LBF from macroalgae by independently measuring oxygen flux rates of algae in isolation. A total of 10 replicates were measured using similar incubation protocols described above, and the average of the four pH and temperature treatment groups was subtracted from the *L. intricata* with *M. vertebralis* replicates to obtain oxygen flux measurements of *M. vertebralis* in association treatment groups (Table 2).

**TABLE 2:** Respiration rates for *Laurencia intricata* in isolation

Treatment groups	Photosynthesis (µmol O_2_ mg *L. intricata* ^−1^)	Respiration (µmol O_2_ mg *L. intricata* ^−1^)	Net production (µmol O_2_ mg *L. intricata* ^−1^)
Ambient temperature, ambient pH	0.221 ± 0.029	0.103 ± 0.012	0.118 ± 0.029
Ambient temperature, low pH	0.174 ± 0.018	0.087 ± 0.005	0.087 ± 0.019
High temperature, ambient pH	0.193 ± 0.023	0.086 ± 0.005	0.107 ± 0.024
High temperature, low pH	0.200 ± 0.023	0.079 ± 0.006	0.121 ± 0.028

Note

These data were used to provide a baseline metabolic rate and subtracted from *Marginopora vertebralis* and *L. intricata* oxygen flux measurements to infer rates of metabolism for *M. vertebralis* in associated treatment groups (see Methods). Values are mean ± *SE* (*n* = 10).

### 2.8 Statistical analyses

For growth rate, instantaneous calcification, maximum photochemical efficiency (*Fv/Fm*), total chlorophyll, and oxygen flux measurement data, a three‐way ANOVA was performed using pH (amb, −0.3 pH units), temperature (amb, and +3°C), and association (no association—treatments of *M. vertebralis* only, and with association—treatments of *M. vertebralis* and *L. intricata*) as fixed factors. Assumptions of ANOVA (homogeneity of variance and normality) were tested and met. All analyses were performed in R Tukey HSD test analyses conducted with the **agricolae** package.

## 3 RESULTS

### 3.1 Growth and calcification parameters

**FIGURE 2:**
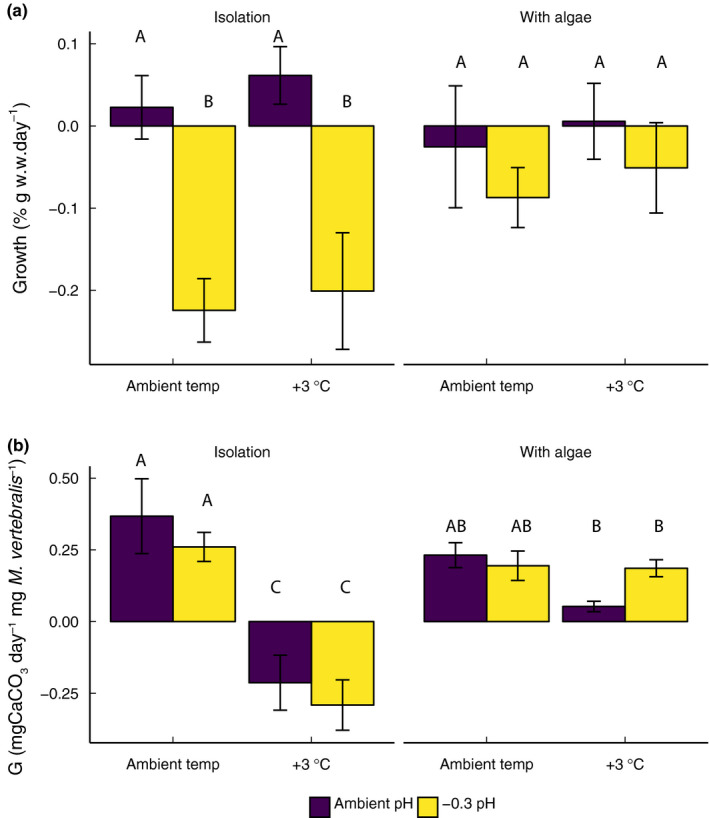
(a) Growth, wet weight change per day with, and (b) calcification (alkalinity anomaly) of *Marginopora vertebralis* incubated in temperature (ambient, +3°C) and pH (ambient, −0.3 pH units) treatments for 2 weeks in isolation and in association with *Laurencia intricata*. Fully factorial mean ± *SE*, *n* = 10 are expressed, and significance is shown with differing letters from Tukey HSD post hoc analyses

A 250% decrease in growth (wet weight change) was observed in *M. vertebralis* in low‐pH conditions when they were not associated with *L. intricata*. In the presence of the algae, growth of *M. vertebralis* was not affected by acidification conditions (*F*_1,72_ = 7.94, *p* < 0.001; Figure 2a; Figure S1; Table S1a). There was no effect of increased temperature on growth rates of *M. vertebralis* regardless of the presence of *L. intricata* (*F*_1,72_ = 2.04, *p* = 0.157; Figure 2a).

Increased temperature caused dissolution of *M. vertebralis* in isolation with *L. intricata*. *Marginopora vertebralis* associated with *L. intricata* had lower calcification compared to associated replicates in ambient pH and temperature conditions, but maintained positive calcification rates in light conditions (*F*_1,72_ = 21.15, *p* < 0.001; Figure 2b; Figure S2; Table S1b).

### 3.2 Holobiont parameters of *Marginopora vertebralis*

**FIGURE 3:**
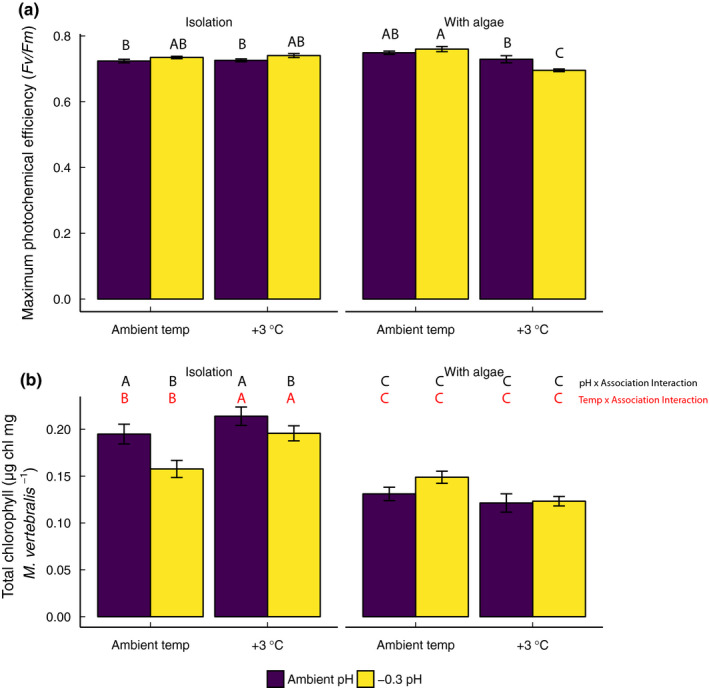
Photophysiological parameters of *Marginopora vertebralis* incubated in temperature (ambient, +3°C) and pH (ambient, −0.3 pH units) treatments for 2 weeks. (a) Maximum photochemical efficiency (*Fv/Fm*) and (b) total chlorophyll content were measured at the end of the experiment. All results are expressed in mean ± *SE*, *n* = 10. Letters indicate Tukey HSD post hoc analyses. Letters represent statistically significant differences

Photochemical efficiency (*Fv/Fm*) of *M. vertebralis* was lowest in acidification and high‐temperature treatments when in association with *L. intricata* (*F*_1,72_ = 7.38, *p* < 0.001; Figure 3a; Table S1c). In contrast, photochemical efficiency was highest in *M. vertebralis* incubated in low pH and ambient temperature when in association with *L. intricata* (*F*_1,72_ = 7.38, *p* < 0.001; Figure 3a; Table S1c).

Total chlorophyll was ~50% higher in *M. vertebralis* in isolation at ambient pH compared with those incubated with *L. intricata* across pH treatments (*F*_1,72_ = 9.92, *p* < 0.001; Figure 3b; Figure S3a). Total chlorophyll was also ~50% higher in *M. vertebralis* incubated at elevated temperatures not in association with *L. intricata* (*F*_1,72_ = 14.93, *p* < 0.001; Figure 3b; Figure S3b; Table S1d) than in controls.

**FIGURE 4:**
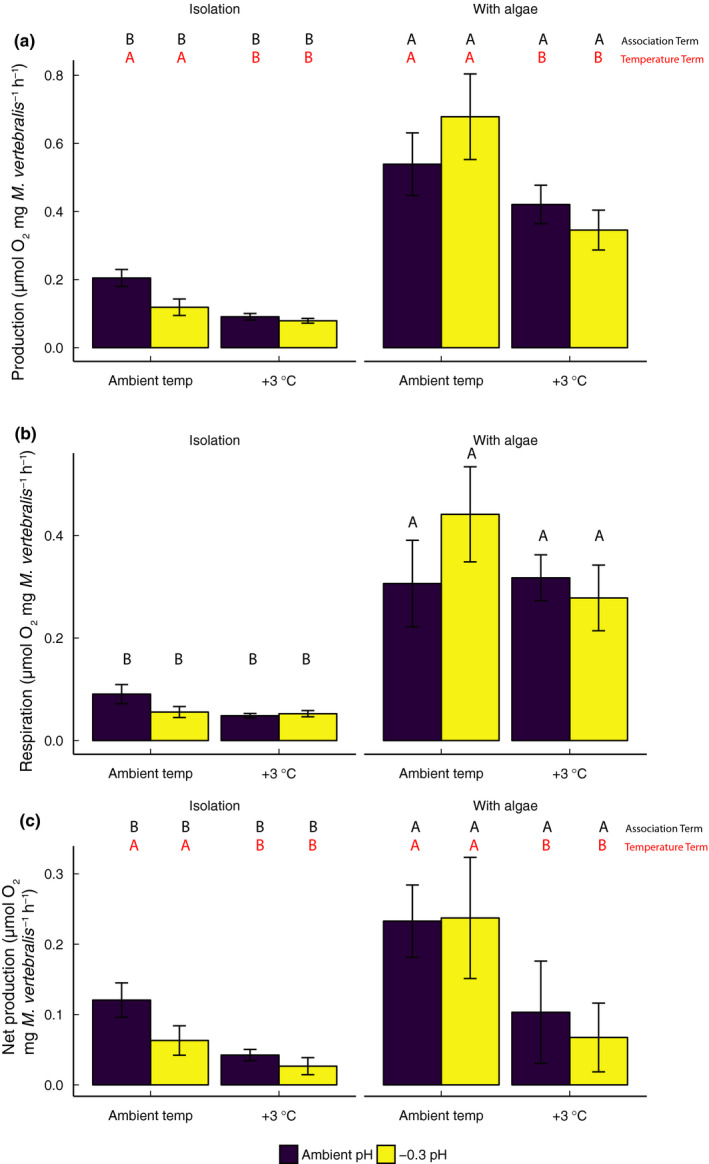
Oxygen flux determined for *Marginopora vertebralis* at the end of the 2‐week experiment in isolation and in association with *Laurencia intricata*. (a) Total production (proxy for photosynthesis) in light (~100 µmol photon m^−2^ s^−1^), (b) respiration (measured in the dark), and (c) net production (the difference between light and dark oxygen fluxes) were measured. All results are expressed as mean ± *SE*, *n* = 10. Differing letters indicate significance using Tukey HSD post hoc analyses

Photosynthetic rates of *M. vertebralis* were significantly lower in high‐temperature treatment groups compared to those in ambient temperatures (*F*_1,72_ = 11.36, *p* < 0.001; Figure 4a; Figure S4a; Table S1e). In addition, photosynthetic rates of *M. vertebralis* in association with *L. intricata* were significantly higher than those incubated in isolation (*F*_1,72_ = 68.91, *p* < 0.001; Figure 4a; Figure S4b).

Respiration rates increased by 80% in *M. vertebralis* associated with *L. intricata* (*F*_1,72_ = 53.79, *p* < .001; Figure 4b; Figure S5), but the main effects of acidification and temperature were not significant and there were no interactions between these factors (see Table S1f). Net oxygen production of associated *M. vertebralis* decreased by 65% in the high‐temperature group (*F*_1,72_ = 9.04, *p* < 0.001; Figure 4b; Figure S6a) and decreased by 60% in isolation (*F*_1,72_ = 7.94, *p* < 0.001; Figure 4c; Table S1g; Figure S6b).

### 3.3 Physiology of *Laurencia intricata* in GCC and OA conditions

Photosynthesis rates of *L. intricata* were not significantly impacted by pH (*F*_1,36_ = 0.75, *p* = 0.390), temperature (*F*_1,36_ = 0.01, *p* = 0.971), or the interaction of these two factors (*F*_1,36_ = 1.30, *p* = .261). Similarly, respiration of *L. intricata* was also not impacted by pH (*F*_1,36_ = 2.41, *p* = 0.129), temperature (*F*_1,36_ = 2.56, *p* = .118), or the interaction of these two factors (*F*_1,36_ = 0.36, *p* = 0.551). Lastly, net photosynthesis of *L. intricata* was not impacted by pH (*F*_1,36_ = 0.12, *p* = .737), temperature (*F*_1,36_ = 0.21, *p* = 0.651), or the interaction of these two factors (*F*_1,36_ = 0.781, *p* = 0.383).

## 4 DISCUSSION

Our study highlights the importance of algae–calcifier relationships as a key component in the resilience of benthic assemblages in the future ocean conditions. In particular, we show the potential for physiological modulation of calcifying organisms to GCC and OA through species interactions with a macroalgae (e.g., Bergstrom et al., 2019; Doo et al., 2018). Consistent with previous studies, we observed decreased growth (wet weight) in response to OA when *M. vertebralis* was incubated in isolation (Doo et al., 2014; Sinutok, Hill, Doblin, Wuhrer, & Ralph, 2011). As seen in previous culturing experiments, this decrease in wet weight is likely an integrated signal of longer‐term (weeks) net dissolution associated with unfavorable carbonate chemistry conditions; however, other effects such as metabolic narcosis could also contribute to decreases in growth observed (Christensen, Nguyen, & Byrne, 2011). *Marginopora* *vertebralis* is also composed of high Mg‐calcite of ~250 mmol/mol (Raja, Saraswati, Rogers, & Iwao, 2005), a mineral form prone to dissolution in OA conditions (Morse, Andersson, & Mackenzie, 2006; Yamamoto et al., 2012). Interestingly, *M. vertebralis* calcification rates (alkalinity anomaly) decreased in response to increased warming only when in isolation. Previous observations of decreases in calcification rate of *M. vertebralis* to GCC show mixed responses of the interactive effects with elevated CO_2_ in which elevated temperature exacerbates OA effects, as determined using through the buoyant weight method (Sinutok et al., 2011), and cross‐sectional surface area changes (Schmidt, Kucera, & Uthicke, 2014). Although we did not see any evidence of pH and temperature interactions on either growth or calcification, it is important to note that calcification measurements were conducted during the day and so nighttime effects were not assessed.

While growth and calcification of *M. vertebralis* were negatively affected by different factors (temperature and CO_2_, respectively), interspecies ecological interactions increased the robustness of *M. vertebralis* to the negative effects of these stressors, maintaining growth and calcification rates in the future climate change scenarios. Previous multiple species investigations in context with climate change have led to interesting observations of physiological buffering, such as increased metabolism of herbivorous fish and associated algae in response to GCC, resulting in increased feeding rates in parallel with maintenance of ecosystem homeostasis in stable fish and algal growth rates (Ghedini et al., 2015), and ectosymbiotic crabs ameliorating the impacts of OA on host scleractinian coral calcification (Doo et al., 2018). At a temperate CO_2_ seep site (analog of projected OA conditions), increased thickness of turf algae is directly linked to increases in calcifier (grazing gastropods) abundance through increased provisioning of suitable habitat and food (Connell et al., 2017). However, studies on LBFs in tropical CO_2_ seep sites show association with seagrass did not ameliorate the negative effects of OA on LBFs (Fabricius et al., 2011; Pettit et al., 2015). As such, a further understanding of biological and ecological interactions between species is a key to understanding climate change impacts in the context of ecological theory (Gaylord et al., 2015).

In studies that have investigated the role of alga–calcifier interactions, photosynthesis of the marine alga is hypothesized to ameliorate the negative effects of OA by increasing pH and saturation state. Although these increases in oxygen production from photosynthesis are demonstrated, DBLs have elevated pH in the micro‐scale due to photosynthesis (Cornwall et al., 2013; Hurd et al., 2011), potentially only benefiting organisms in close association with the algal basiphyte (Semesi, Beer, & Björk, 2009). Although we did not directly test for DBLs in this study, previous results in addition to other photophysiological parameters of total chlorophyll indicate that *L. intricata* may provide a stable refugium for associated LBFs due to production of a large DBL (Borowitzka, Larkum, & Borowitzka, 1978). *Marginopora vertebralis* also produces a large DBL (Glas & Fabricius, 2012) suggesting a shift in nutrient acquisition strategy from actively acquiring organic carbon resources through photosynthesis to passive environmental acquisition of similar resources maintaining homeostasis (calcification rate) in association with *L. intricata*. *Marginopora vertebralis* is also known to exhibit flexibility in biochemical responses to OA, allowing for acclimatization to changing ocean conditions (Prazeres, Uthicke, & Pandolfi, 2015). Further, a decrease in total chlorophyll levels of *M. vertebralis* was observed when in association with *L. intricata*, while increasing maximum photochemical efficiency at ambient temperatures. These results suggest that photo‐oxidative stress from endosymbiont photosynthesis for the LBF holobiont could be limited through adaptation mechanisms of decrease in total chlorophyll in conjunction with increased maximum photochemical efficiency (Prazeres, Uthicke, & Pandolfi, 2016). The increased stability that is gained through living on algae may lead to more favorable conditions, in which decreased total chlorophyll levels of *M. vertebralis* in association with *L. intricata* are able to maintain similar calcification rates (Prazeres et al., 2016). These shifts in physiological responses of *M. vertebralis* acquired through interaction with *L. intricata* highlight that LBFs may be adapted to algal substrata, and have the potential to use this interaction to buffer against changing ocean conditions.

In the current study, compensatory responses of increases in chl‐a in *M. vertebralis* cultured in isolation are observed in tandem with physiological changes resulting from ecological interactions (amelioration of negative OA and GCC impacts on growth and calcification when cultured with *L. intricata*). The effects of OA are, however, buffered when the LBF is associated with *L. intricata*, where pH increased during the day due to photosynthesis. While nighttime respiration decreases the pH of the micro‐scale boundary layer of algae in which LBFs reside, our results indicate that *M. vertebralis* cultured with *L. intricata* exhibited similar overall growth rates compared with that observed in ambient conditions. The diel migration patterns have been observed for *M. vertebralis* at night to the surface of algal beds and have been attributed to the avoidance of chlorophyll degradation and photoinhibition during daytime (Sinutok, Hill, Doblin, & Ralph, 2013). However, this behavioral response may also be due to the migration away from hypoxic and more acidic nighttime conditions within the algal substratum microenvironment due to algal respiration (Kapsenberg & Cyronak, 2019). In the current study, most of the *M. vertebralis* were found attached to the apical regions of *L. intricata* throughout day–night, suggesting light conditions within our experiment were not sufficient to induce photoinhibition (Sinutok et al., 2013); however, association with the algal substrata also has the potential to provide shading that could influence organismal physiology of LBFs. In addition, *M. vertebralis* is known to feed on detritus which may influence its location in the algal substrata (ter Kuile, Lee, & Anderson, 1991).

The results of this study have important implications with respect to our understanding of calcification rates of common high carbonate‐producing LBFs. Carbonate production rates of calcifiers such as *M. vertebralis* have documented high rates of calcification and contribution to reef‐scale accretion (Doo et al., 2016), but such measurements of calcification rates are often taken using isolated specimens without the associated algal substrata or commonly associated organisms. This study suggests that calcification rates may differ drastically in calcifiers incubated in isolation versus in ecologically relevant scenarios in association with their natural substrata, due to biological buffering from the algal DBL. In addition, our study indicates that the substratum choice is likely a key factor in the survival of *M. vertebralis* in a changing ocean, as the status of calcifiers that live in isolation from algal substrata will be impaired compared to those living in association with certain algal habitats. Species interactions, particularly multispecies symbioses that provide biological buffering services, are an important ecological process that needs further investigation to more accurately estimate the mechanistic changes that may occur under future climate change scenarios (Doo et al., 2020).

## CONFLICT OF INTEREST

None declared.

## AUTHOR CONTRIBUTIONS

**Steve S. Doo:** Conceptualization (lead); data curation (lead); formal analysis (lead); funding acquisition (lead); investigation (lead); methodology (lead); project administration (lead); resources (equal); validation (lead); visualization (lead); writing – original draft (lead); writing – review & editing (equal). **Aero Leplastrier:** Conceptualization (equal); data curation (supporting); investigation (equal); methodology (equal); validation (equal); writing – review & editing (equal). **Alexia Graba‐Landry:** Conceptualization (equal); data curation (equal); formal analysis (supporting); investigation (equal); methodology (supporting); writing – review & editing (equal). **Januar Harianto:** Conceptualization (equal); data curation (equal); formal analysis (equal); methodology (equal); writing – review & editing (equal). **Ross A. Coleman:** Conceptualization (equal); methodology (equal); supervision (supporting); writing – review & editing (equal). **Maria Byrne:** Conceptualization (equal); formal analysis (equal); funding acquisition (equal); methodology (equal); project administration (equal); supervision (lead); writing – original draft (equal); writing – review & editing (equal).

## Supporting information

Fig S1‐S6

Table S1

## Data Availability

All data included in this manuscript have been included in the supporting information file and have been collated and deposited on the Dryad repository https://doi.org/10.5061/dryad.qv9s4mwbw.
