# Supplementary material for: Amelioration of ocean acidification and warming effects through physiological buffering of a macroalgae
Source: Ecol Evol. 2020 Jul 19;10(15):8465–75. doi: 10.1002/ece3.6552 (PMC7417211; doi:10.1002/ece3.6552)

Supplementary Figures 1-6

Supplementary Figure 1. Mean ± S.E. values of growth rate of *Marginopora vertebralis* grouped according to statistically significant factors of pH treatments (ambient, -0.3 pH units), and Association (isolation and with algae).
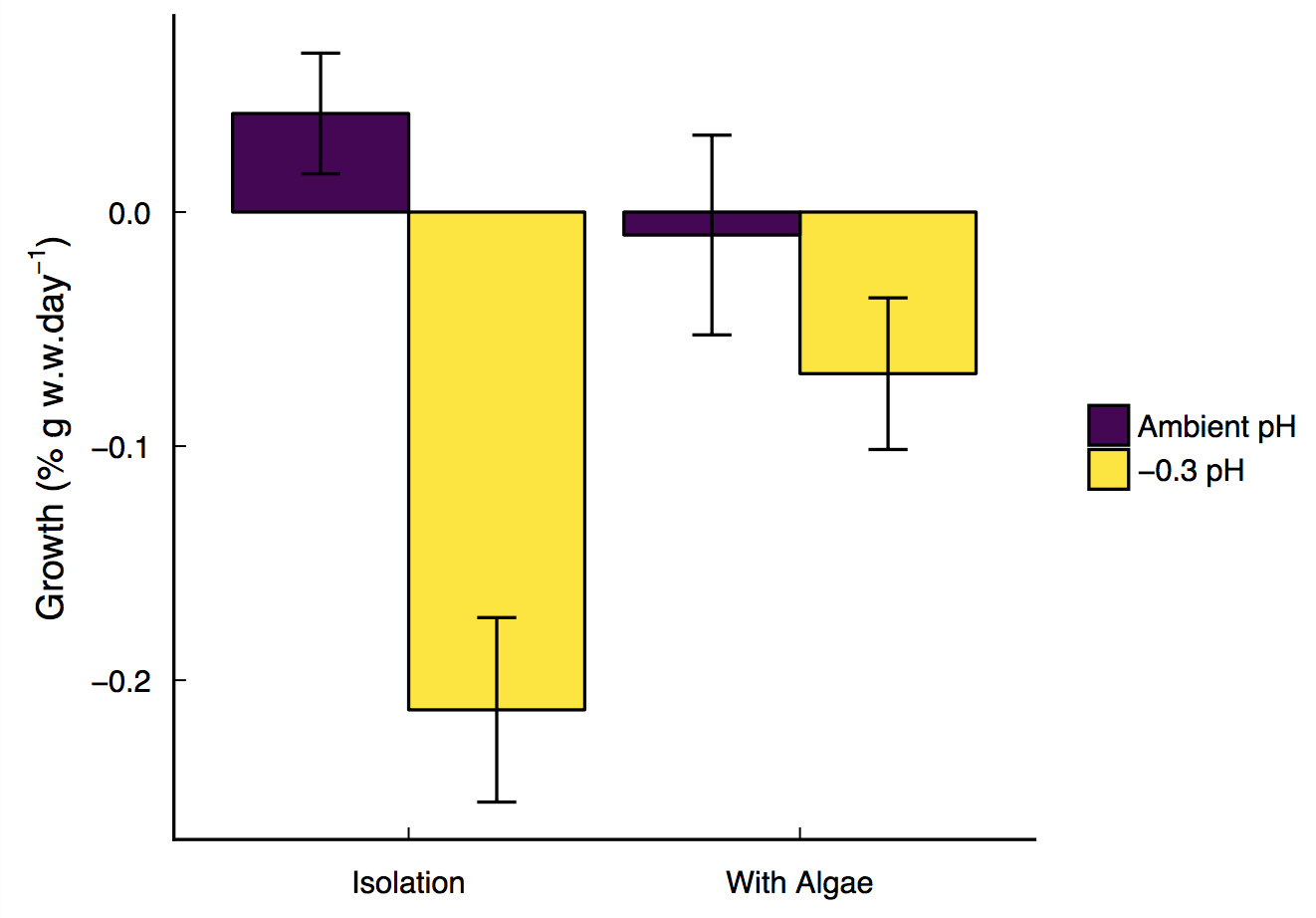


Supplementary Figure 2. Mean ± S.E. values of calcification rate of *Marginopora vertebralis* grouped according to statistically significant factors of temperature treatments (ambient, +3°C), and Association (isolation and with algae).


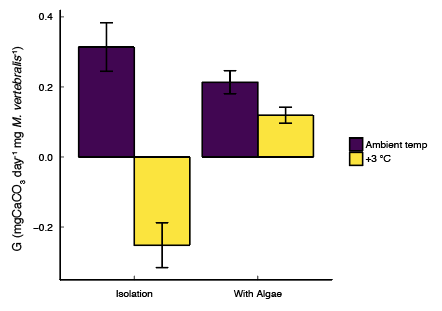


Supplementary Figure 3. Mean ± S.E. values of total chlorophyll of *Marginopora vertebralis* grouped according to statistically significant factors of **(A)** pH treatments (ambient, -0.3 pH units), and Association (isolation and with algae), and **(B)** temperature treatments (ambient, +3°C) and Association (isolation and with algae).
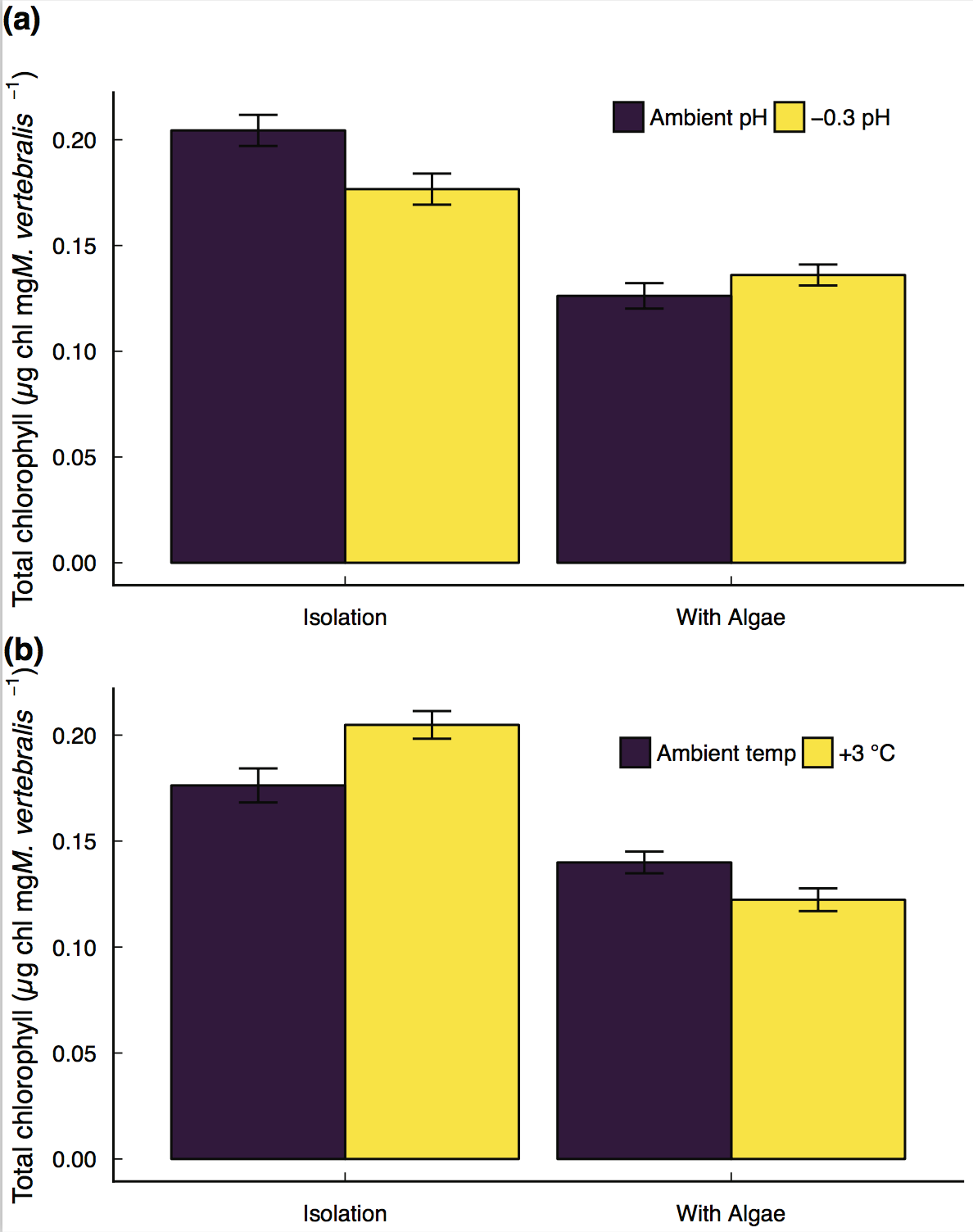


Supplementary Figure 4. Mean ± S.E. values of production (photosynthesis) of *Marginopora vertebralis* grouped according to statistically significant factors of **(A)** temperature treatments (ambient, +3°C), and **(B)** pH treatments (ambient, -0.3 pH units).
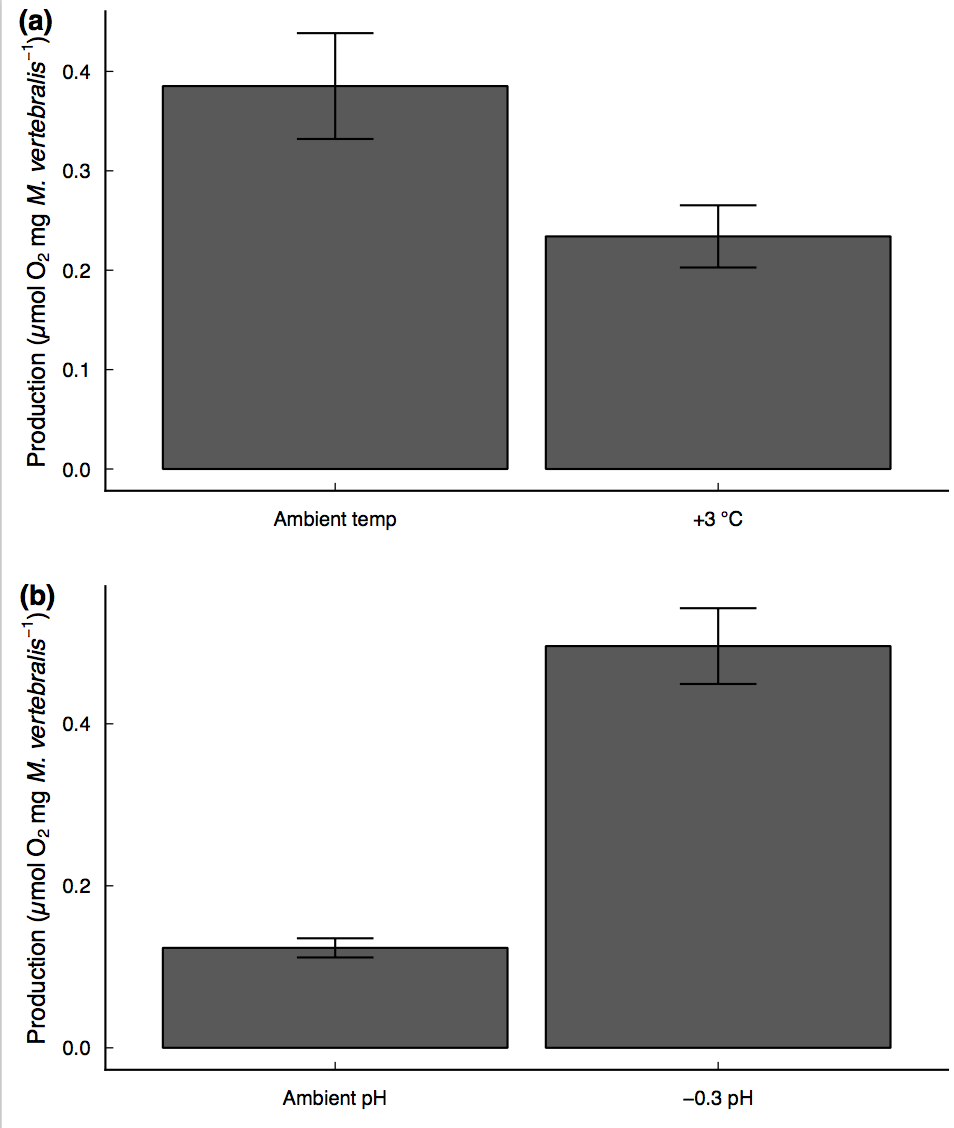


Supplementary Figure 5. Mean ± S.E. values of respiration of *Marginopora vertebralis* grouped according to the statistically significant factor of association (isolation and with algae).
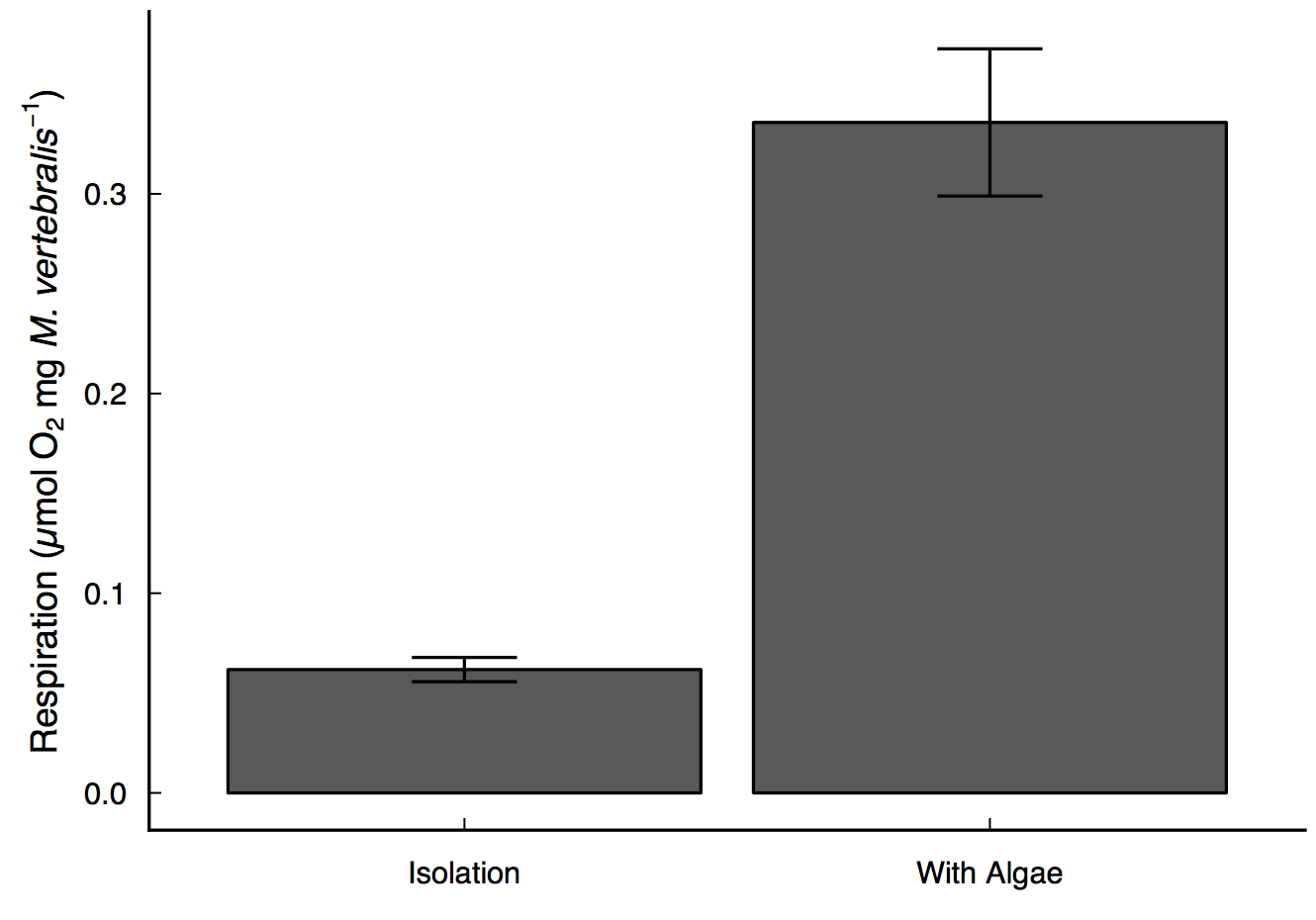


Supplementary Figure 6. Mean ± S.E. values of net production (photosynthesis and respiration) of *Marginopora vertebralis* grouped according to statistically significant factors of **(A)** temperature treatments (ambient, +3°C), and **(B)** Association treatments (isolation and with algae).


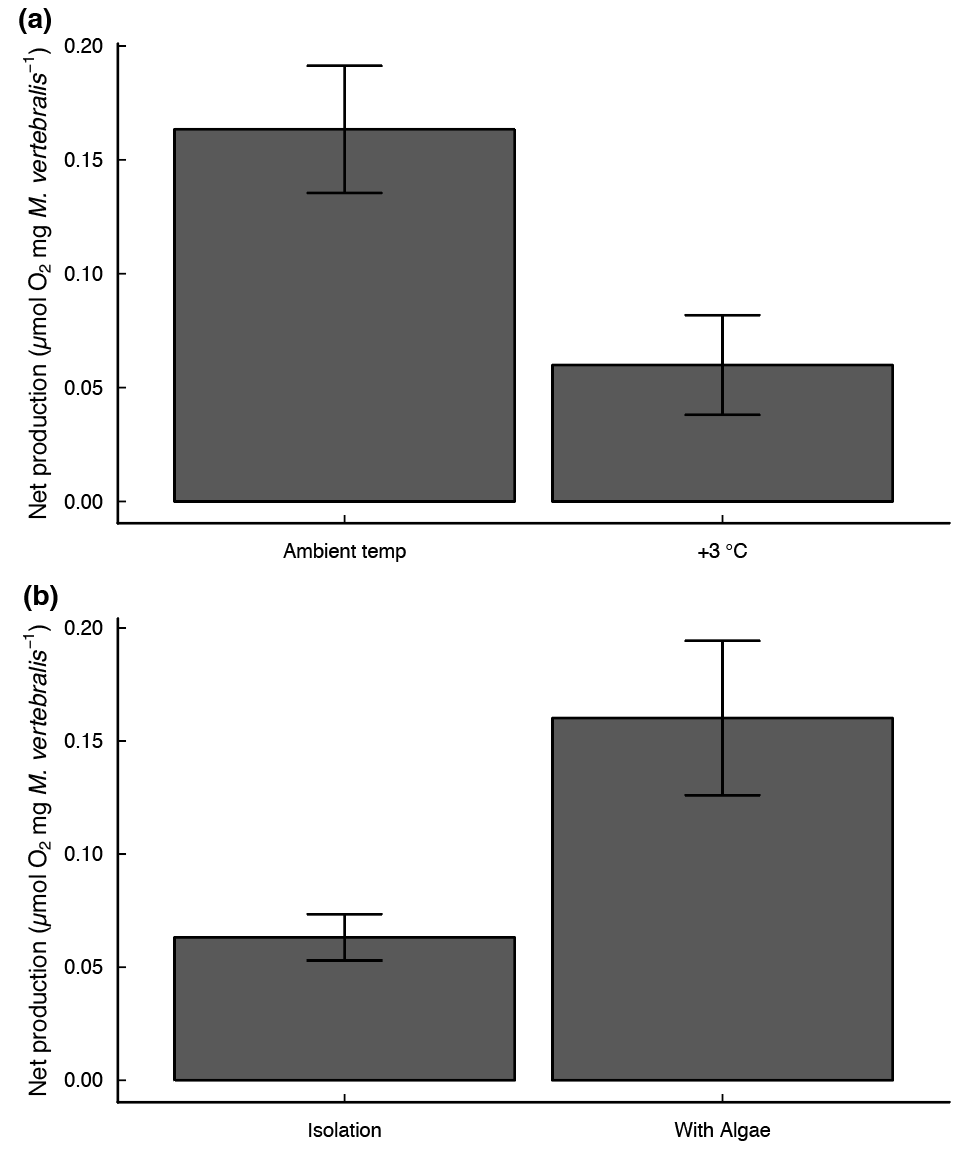

Supplement: Supplementary file 1 — Fig S1‐S6 [file ECE3-10-8465-s001.docx]
