# Supplementary material for: Amelioration of ocean acidification and warming effects through physiological buffering of a macroalgae
Source: Ecol Evol. 2020 Jul 19;10(15):8465–75. doi: 10.1002/ece3.6552 (PMC7417211; doi:10.1002/ece3.6552)
Supplement: Supplementary file 2 — Table S1 [file ECE3-10-8465-s002.docx]

Supplementary files

Supplementary Table 1. ANOVA results for all analyses. All analyses can be found in the Methods.

| **A. Growth (% Change in weight day^-1^)** | |  |  |  | |  | |
| --- | --- | --- | --- | --- | --- | --- | --- |
| Source | df | MS | F | p | | Tukey HSD | |
| pH | 1 | 0.49 | 18.57 | **<0.01** | | amb>low | |
| Temperature | 1 | 0.02 | 0.79 | 0.16 | |  | |
| Association | 1 | 0.04 | 1.58 | 0.25 | |  | |
| pH*Temperature | 1 | <0.01 | 0.01 | 0.55 | |  | |
| pH*Association | 1 | 0.19 | 7.20 | **0.01** | | See below^1^ | |
| Temperature*Association | 1 | <0.01 | <0.01 | 0.88 | |  | |
| pH*Temperature*Association | 1 | <0.01 | 0.02 | 0.98 | |  | |
| Error | 72 | 0.03 |  |  | |  | |
| Total | 79 |  |  |  | |  | |
| ^1^Amb, Foram (A); Amb, Both (A); -0.4 pH, Both (A); -0.4 pH, Foram (B) | | | |  | |  | |
|  |  |  |  |  | |  | |
| **B. Calcification rate** |  |  |  |  | |  | |
| Source | df | MS | F | p | | Tukey HSD | |
| pH | 1 | 0.01 | 0.19 | 0.67 | |  | |
| Temperature | 1 | 2.18 | 41.30 | **<0.01** | | Amb>High | |
| Association | 1 | 0.37 | 6.95 | **0.01** | | Yes > No | |
| pH*Temperature | 1 | 0.05 | 0.94 | 0.33 | |  | |
| pH*Association | 1 | 0.10 | 1.88 | 0.17 | |  | |
| Temperature*Association | 1 | 1.11 | 21.15 | **<0.01** | | See below^1^ | |
| pH*Temperature*Association | 1 | 0.02 | 0.47 | 0.49 | |  | |
| Error | 72 | 0.05 |  |  | |  | |
| Total | 79 |  |  |  | |  | |
| ^1^Amb, Foram (A); Amb, Both(AB); +4°C, Both (B); +4°C, Foram '(C) | | | | | | |  |
|  | | | | | | |  |
|  |  |  |  |  | |  | |
| **C. Maximum photochemical efficiency (*Fv/Fm*)** | | |  |  | |  | |
| Source | df | MS | F | p | | Tukey HSD | |
| pH | 1 | 1.1 E-5 | 0.03 | 0.87 | |  | |
| Temperature | 1 | 7.2 E-3 | 18.40 | **<0.01** | |  | |
| Association | 1 | 1.0 E-4 | 0.26 | 0.61 | |  | |
| pH*Temperature | 1 | 2.1 E-3 | 5.30 | **0.02** | |  | |
| pH*Association | 1 | 2.9 E-3 | 7.26 | **0.01** | |  | |
| Temperature*Association | 1 | 0.01 | 26.80 | **<0.01** | |  | |
| pH*Temperature*Association | 1 | 2.9 E-3 | 7.38 | **0.01** | | See below^1^ | |
| Error | 72 | 4.0 E-4 |  |  | |  | |
| Total | 79 |  |  |  | |  | |
| ^1^-0.4 pH, amb ,Algae (A); Amb, Amb, Algae (AB); -0.4 pH, +4°C, Foram (AB); -0.4 pH, Amb, Foram (AB); Amb, +4°C, Algae (B); Amb, +4°C, Foram (B); Amb, Amb, Foram (B); -0.4 pH, +4°C, Algae "(C)" | | | | | | |  |
|  |  |  |  |  |  |  |  |
|  |  |  |  |  | |  | |
| **D. Total Chlorophyll** |  |  |  |  | |  | |
| Source | df | MS | F | p | | Tukey HSD | |
| pH | 1 | 1.6 E-3 | 2.24 | 0.14 | |  | |
| Temperature | 1 | 6.0 E-4 | 0.84 | 0.36 | |  | |
| Association | 1 | 0.07 | 98.93 | **<0.01** | | No>Yes | |
| pH*Temperature | 1 | 1.0 E-5 | 0.02 | 0.90 | |  | |
| pH*Association | 1 | 0.01 | 9.92 | **<0.01** | | See below^1^ | |
| Temperature*Association | 1 | 0.01 | 14.93 | **<0.01** | | See below^2^ | |
| pH*Temperature*Association | 1 | 1.5 E-3 | 2.13 | 0.15 | |  | |
| Error | 72 | 7.1 E-4 |  |  | |  | |
| Total | 79 |  |  |  | |  | |
| 1Amb, No (A);-0.4 pH, No (B); -0.4pH, Yes (C); Amb, Yes (C) | | | | |  | |  |
| 2+4°C, Foram (A); Amb, Foram (B); Amb, Both (C); +4°C, Both (C) | | | |  | |  | |
|  |  |  |  |  | |  | |
| **E. Production** |  |  |  |  | |  | |
| Source | df | MS | F | p | | Tukey HSD | |
| pH | 1 | 1.4 E-3 | 0.04 | 0.85 | |  | |
| Temperature | 1 | 0.46 | 11.36 | **<0.01** | | amb>high temp | |
| Association | 1 | 2.78 | 68.91 | **<0.01** | | Yes > No | |
| pH*Temperature | 1 | 0.02 | 0.61 | 0.44 | |  | |
| pH*Association | 1 | 0.03 | 0.82 | 0.37 | |  | |
| Temperature*Association | 1 | 0.11 | 2.75 | 0.10 | |  | |
| pH*Temperature*Association | 1 | 0.10 | 2.58 | 0.11 | |  | |
| Error | 72 | 0.04 |  |  | |  | |
| Total | 79 |  |  |  | |  | |
|  |  |  |  |  | |  | |
| **F. Respiration** |  |  |  |  | |  | |
| Source | df | MS | F | p | | Tukey HSD | |
| pH | 1 | 5.2 E-3 | 0.19 | 0.67 | |  | |
| Temperature | 1 | 0.05 | 1.74 | 0.19 | |  | |
| Association | 1 | 1.50 | 53.79 | **<0.01** | | Yes > No | |
| pH*Temperature | 1 | 0.02 | 0.82 | 0.37 | |  | |
| pH*Association | 1 | 0.02 | 0.72 | 0.40 | |  | |
| Temperature*Association | 1 | 0.01 | 0.51 | 0.48 | |  | |
| pH*Temperature*Association | 1 | 0.06 | 2.03 | 0.160 | |  | |
| Error | 72 | 0.03 |  |  | |  | |
| Total | 79 |  |  |  | |  | |
|  |  |  |  |  | |  | |
|  |  |  |  |  | |  | |
| **G. Net Production** |  |  |  |  | |  | |
| Source | df | MS | F | p | | Tukey HSD | |
| pH | 1 | 0.01 | 0.58 | 0.45 | |  | |
| Temperature | 1 | 0.21 | 9.04 | **<0.01** | | Amb > High | |
| Association | 1 | 0.19 | 7.94 | **<0.01** | | Yes > No | |
| pH*Temperature | 1 | 1.0 E-6 | <0.01 | 0.99 | |  | |
| pH*Association | 1 | 2.2 E-3 | 0.09 | 0.76 | |  | |
| Temperature*Association | 1 | 0.04 | 1.80 | 0.18 | |  | |
| pH*Temperature*Association | 1 | 8.4 E-3 | 0.35 | 0.55 | |  | |
| Error | 72 | 0.02 |  |  | |  | |
| Total | 79 |  |  |  | |  | |
